# Process evaluation of a programme to empower community nurse leadership

**DOI:** 10.1186/s12912-021-00650-y

**Published:** 2021-07-12

**Authors:** Ruth G. M. Vogel, Gerrie J. J. W. Bours, Teuni H. Rooijackers, Silke F. Metzelthin, Petra M. G. Erkens, Erik van Rossum, Sandra M. G. Zwakhalen

**Affiliations:** 1 Department of Health Services Research, Maastricht University, Care and Public Health Research Institute, Maastricht, The Netherlands; 2 Living Lab in Ageing and Long-Term Care, Maastricht, The Netherlands; 3 Research Centre for Community Care, Academy of Nursing, Zuyd University of Applied Sciences, Heerlen, The Netherlands

**Keywords:** Leadership, Community health nurses, Implementation science, Evaluation

## Abstract

**Background:**

The Nurses in the Lead (NitL) programme consists of a systematic approach and training to 1) empower community nurses in implementing evidence, targeted at encouraging functional activities of older adults, and 2) train community nurses in enabling team members to change their practice. This article aims to describe the process evaluation of NitL.

**Methods:**

A mixed-methods formative process evaluation with a predominantly qualitative approach was conducted. Qualitative data were collected by interviews with community nurses (*n* = 7), focus groups with team members (*n* = 31), and reviewing seven implementation plans and 28 patient records. Quantitative data were collected among community nurses and team members (*N* = 90) using a questionnaire to assess barriers in encouraging functional activities and attendance lists. Data analysis was carried out through descriptive statistics and content analysis.

**Results:**

NitL was largely executed according to plan. Points of attention were the use and value of the background theory within the training, completion of implementation plans, and reporting in patient records by community nurses. Inhibiting factors for showing leadership and encouraging functional activities were a lack of time and a high complexity of care; facilitating factors were structure and clear communication within teams. Nurses considered the systematic approach useful and the training educational for their role. Most team members considered NitL practical and were satisfied with the coaching provided by community nurses. To optimise NitL, community nurses recommended providing the training first and extending the training. The team members recommended continuing clinical lessons, which were an implementation strategy from the community nurses.

**Conclusions:**

NitL was largely executed as planned, and appears worthy of further application in community care practice. However, adaptations are recommended to make NitL more promising in practice in empowering community nurse leadership in implementing evidence.

**Supplementary Information:**

The online version contains supplementary material available at 10.1186/s12912-021-00650-y.

## Background

Community-based care provision is becoming more complex due to an ageing society and the rising number of older adults with chronic diseases [1]. In this increasingly challenging context, community nurses are seen as key players within the Dutch community care setting [2]. These nurses are bachelor-educated and in charge of a community care team consisting of 10–15 registered nurses, certified nurse assistants and helping aids. Community nurses are at the forefront of improving quality of care and leading their team members throughout this process in practice [3]. In this study, we define nurse leadership as “a process where nurses can develop observable leadership competencies and attributes needed to improve patient outcomes, personnel outcomes and organisational outcomes” [4]. Their leadership role enables them to implement evidence into community care and contribute to enhancing patient outcomes [3].

The Nurses in the Lead (NitL) programme was designed to empower community nurse leadership in implementing evidence, targeted at encouraging functional activities of older adults [5]. Empowerment in this context means that community nurses are strengthened in their leadership role – to implement specific evidence within community care, and lead their team members throughout this process. By empowering their leadership, more evidence for encouraging functional activities of older adults may be implemented in practice. Performing functional activities independently, for example (instrumental) activities of daily living ([I]ADL), can maintain functional independence and autonomy of older adults [6, 7]. However, nurses are traditionally familiar with completing care activities for older adults [8–10] and, therefore, it is key to implement evidence for encouraging functional activities of older adults.

The NitL programme entails two components [5]. First, a systematic approach based on the Implementation of Change Model [11] to empower nurses in implementing evidence. This approach contains six implementation steps and five implementation tools, which can guide them in systematically implementing evidence, provided via an e-learning programme. The second component is group training to empower nurses in enabling team members to change their practice, focusing on motivational interviewing [12], dealing with resistance to change, and the coaching of teams [13–15].

We evaluated NitL’s outcomes and conducted a process evaluation. The evaluation of the outcomes is reported elsewhere [16], showing that the programme was perceived as valuable and may lead to positive impacts for strengthening leadership. The current study reports on process evaluation, according to the framework of Saunders [17], and aims to evaluate the process of implementing NitL in practice. The following overall research question and sub-questions guided the study: How was the NitL programme implemented into community care practice? The following sub-questions were formulated to help answer this overall question:

A. To what extent are the components of NitL delivered and received?
B. To what extent is attendance achieved?
C. To what extent is the programme implemented as planned?
D. To what extent is satisfaction with the programme experienced?
E. To what extent were barriers and facilitators encountered while implementing NitL?

## Methods

### Context

NitL was delivered in seven community care teams from two long-term care organisations, providing community care to patients. The organisations consist of several community care teams that each comprise 10–15 team members and one community nurse. The bachelor-educated community nurse is in charge of the team members, who can either be registered nurses with a bachelor’s degree or vocational training, or certified nurse assistants or helping aids with a secondary training. These teams provide personal care such as washing, and nursing care such as wound treatment. The community nurse determines what and how much care is needed for clients, while taking prevention, tailored care and advice into account [18].

### Design

A mixed-methods formative process evaluation, with a predominantly qualitative approach, was conducted during February 2018–January 2019, following the conventional [19, 20] process indicators of Saunders et al. [17]. NitL was implemented in three consecutive rounds of 8 months. A formative evaluation was conducted to anticipate implementation difficulties during subsequent rounds, and possibly adjust the NitL programme. Formative evaluations are often designed using mixed methods, to be able to gain a deeper understanding of how a programme is implemented [21–23]. By combining qualitative and quantitative methods, we were able to provide broader insights on the implementation of NitL. We could ensure that quantitative results were combined with the experiences of community nurses and team members, and improve our understanding of the components of NitL in practice [24]. Qualitative data were collected via interviews with community nurses, focus group interviews with their team members, and reviews of implementation plans and patient records. Quantitative data were collected by a questionnaire to assess barriers in encouraging functional activities among community nurses and team members [25] and a review of attendance lists. The study is reported following the Good Reporting of a Mixed Methods Study (GRAMMS) [26].

### Setting and sample

Seven bachelor-educated community nurses from two community care organisations were recruited via convenience sampling to participate. Purposeful sampling was used to recruit a minimum of 14 team members (at least two per community care team) to participate in the focus group interviews based on variations in demographic characteristics (profession, education, work experience, specific focus area within the team, and work hours per week). The seven community nurses and all 83 team members were invited to participate in the quantitative data collection. In addition, 28 patient records of older adults were purposefully sampled, based on selection criteria of being aged over 75 years and receiving community nursing care by one of the community care teams.

### The NitL programme

The development and content of NitL is described in more detail elsewhere [5]. The programme was based on the learning needs of seven community nurses, who also participate in the current study. The first component of NitL, the systematic approach, consists of six implementation steps that nurses can use to develop an implementation plan. For example, in the first step, nurses are guided in developing a proposal for change. To complete the steps, they can use several practical implementation tools, such as a format for developing the implementation plan and the Maastricht Nurses Activities Inventory for Community care (MAINtAIN-C) questionnaire, to measure the perceived behaviour and barriers of their team members in encouraging functional activities [25]. The second component is training to empower community nurses in enabling team members to change practice. The training is a blend of 4-h face-to-face group training and background theory offered via e-learning. It addresses motivational interviewing [12], dealing with resistance to change and coaching care teams, for example during peer supervision meetings [13–15]. During the first 2 months of the implementation, nurses developed an implementation plan by making use of the e-learning (i.e., the systematic approach). Implementation tools could be used to complete the plan. Two or three community nurses together developed an implementation plan, and received support via bimonthly meetings with an interventionist (author RGMV) experienced in implementation processes and with a nursing background. During the following 6 months, the nurses implemented their plan in practice and had monthly meetings with the interventionist to evaluate the process. They also received group training at this time (Web-based e-learning was constantly available.)

### Measurement instruments and data collection

We assessed the process indicators dose delivered, dose received exposure, reach, fidelity, dose received satisfaction and context, according to the framework of Saunders et al. [17]. Table 1 provides insight into the operationalisation of the components and data collection methods.

**Table 1:** Outcome measures, operationalisation and data collection of the process evaluation

Measures	Operationalisation	Data collection methods
**Dose delivered**	The extent to which the components of the NitL programme, namely the systematic approach and training, were delivered to community nurses and all intended content of the programme covered.	• Interviews with community nurses
**Dose received exposure**	The extent to which community nurses actively engaged with and used the systematic NitL approach and training.	• Interviews with community nurses
**Reach**	The proportion of community nurses who attended plenary meetings during the implementation of NitL.	• Attendance lists
**Fidelity**	The extent to which NitL was implemented as planned, related to 1) how community nurses implemented evidence for encouraging functional activities and enabled team members to change practice, and 2) how team members were enabled by community nurses to encourage functional activities.	• Interviews with community nurses • Focus groups with team members • Patient records • Implementation plans
**Dose received satisfaction**	The satisfaction of 1) community nurses with the programme related to implementing evidence for encouraging functional activities, and enabling team members to change practice, and 2) team members, related to encouraging functional activities and how they were enabled to change practice by community nurses.	• Interviews with community nurses • Focus groups with team members
**Context**	The extent to which barriers and/or facilitators were encountered while implementing the NitL programme related to 1) community nurses while implementing evidence and enabling team members to change practice, and 2) team members while encouraging functional activities and being enabled to change practice by community nurses.	• Interviews with community nurses • Focus groups with team members • MAINtAIN-C questionnaire

#### Qualitative data collection

Interviews with community nurses were conducted by one researcher (author RGMV) during the sixth month of implementation and 1 month after the implementation. Focus group interviews with team members were conducted 1 month after implementation by two researchers (authors RGMV and GJJWB). The topic lists for the interviews were based on the components of Saunders et al. [17] (for more details see Additional file 1). Data saturation level was reached (enough in-depth data was available [27]) after 6 interviews with nurses (2 with nurses in the first round, 2 in the second round, and 2 in the third) and three focus groups with team members. To review implementation plans, a checklist was developed (see Additional file 2) based on the Implementation of Change Model [11] to assess whether the plans matched given implementation steps within the systematic approach. The plans were reviewed in January 2019 by two researchers (authors GJJWB and PMGE).

To review the patient records, a checklist was developed (see Additional file 3) to assess whether nursing diagnoses, interventions and outcomes related to encouraging functional activities (ADL, IADL) were described by community nurses and reported by their team members. Patient records were retrospectively reviewed in January 2019 by one researcher (author RGMV) looking at the fifth, sixth, seventh and eighth month of each implementation round.

#### Quantitative data collection

The MAINtAIN-C Barriers scale [25] was completed by community nurses and their team members. The scale measures their perceived barriers in encouraging functional activities, with seven items related to clients’ context (α = .78) and 21 items related to the context of professionals, the social and organisational context (α = .83). In an earlier study, the scale was adapted from the MAINtAIN scale for nursing homes by the same seven community nurses as participated in this study [25]. The scale was sent to the community nurses and the nurses in their teams 1 month after the implementation via an online programme. Between 2 and 4 weeks after the initial invitation, reminder emails were sent to the non-responders. Further, background characteristics (e.g. age and years of work experience) of community nurses and team members were assessed via the scale. Attendance lists were used in support meetings with the interventionist, and in group training.

### Data analysis

#### Qualitative data analysis

Interviews and focus groups were audiotaped and transcribed verbatim. NVivo 11 was used as supportive software [28]. Based on the principles of directed content analyses [29], the topic lists were used as a guiding analytical framework to analyse the data. At first, data were coded following the topics, by author RGMV. A second author (GJBB) verified the developed codes by looking at text, codes and topics. This was discussed during consensus meetings with authors RGMV and GJJB. Differences in interpretation were solved by dialogue to reach consensus. Subsequently, two authors (RGMV and THR) independently grouped the earlier developed codes until sub-categories emerged. Any differences in interpretation were discussed until consensus was reached. Finally, one researcher (author GJJB) verified the categories and made minor adaptations. A professional native-speaker translated the quotes into English. For the review of implementation plans, two researchers (authors GJJB and PMGE) independently assessed if each step was described completely, partly, or not at all. This was discussed with one researcher (author RGMV) and any discrepancies in scoring were resolved until consensus was reached. For the review of patient records, one author (RGMV) analysed if and which nursing diagnoses, interventions and outcomes were described and reported in the records. The analysis of the first four records was discussed with another researcher (GJJWB) to reach consensus.

#### Quantitative data analysis

Quantitative data were analysed using IBM SPSS Statistics 25.0 for Windows [30]. Descriptive statistics were used to present the characteristics of the study sample. For the MAINtAIN-C Barriers scale, missing values were imputed based on the average score of all respondents on all items for the team members, or with the average score of all respondents on the missing item for the community nurses, if at least 80% of items had been completed. Those missing more than 20% were excluded from the analyses. The positively formulated items were reversed, to make sure that higher scores indicate stronger experienced barriers. Further, descriptive statistics were used to develop an overview of attendance.

### Synthesis of mixed methods

After separate analysis of quantitative and qualitative data, findings were merged following the convergent mixed-methods design [24]. A side-by-side analysis was undertaken by discussing first the qualitative findings and then quantitative results. In this way, both qualitative data (views of community nurses and their team members) and quantitative data (from the questionnaire) provided a complete understanding [24].

### Trustworthiness

Several strategies were used to meet the criteria of credibility, transferability and confirmability [31] to enhance the trustworthiness of the study [32]. First, credibility was enhanced by triangulation of investigators and data. Triangulation of investigators involved a reflection of all the authors on the design, collection and analyses of the study. Furthermore, the coding, analysing and interpreting of data were completed by two researchers. Triangulation of data was achieved by including different sources within the study (multiple respondents in focus groups and multiple interviews). To enhance confirmability, two researchers performed the qualitative data analysis.

## Results

### Sample characteristics

Table 2 provides baseline characteristics of the seven community nurses, 31 team members who participated in three focus groups (with, respectively, 10, 11 and 10 participants) and 69 team members who completed the MAINtAIN-C questionnaire. Missing data on the MAINtAIN-C questionnaire were due to sickness, absence or leaving the team. For the review of patient records, 28 older adults with a median age of 82 years (*SD* = 5.5) gave consent to participate; 16 were female.

**Table 2:** Sample characteristics of community nurses (*n* = 7) and team members that participated in the focus groups (*n* = 31) and completed the questionnaire (*n* = 69)^a^

	Community nurses *(n = 7)*		Team members *(n = 31)*		Team members*(n = 69)*	
	*n*	%	*n*	%	*n*	%
Gender						
Female	6	85.7	30	96.8	67^b^	97.1
Profession						
Bachelor-educated nurse	7	100.0	4	12.9	9^b^	13.0
Vocationally educated nurse			9	29.0	13	18.8
Certified nurse Assistant/Helping aid/Nursing student			18	58.1	45	65.2
Education						
Master of science	2	28.6			9^c^	13.0
Bachelor of nursing	5	71.4	5	16.1	18	26.1
Vocational training			11	35.5	38	55.1
Secondary training			15	48.4		
	Median	Range [min-max]	Median	Range [min-max]	Median	Range [min-max]
Age in years	34	31 [26–56]	51	12.1 [22–61]	49^b^	43 [21–64]
Work experience in years	14	31 [7–38]	22	11.0 [5–41]	19^c^	47 [1–47]
Working hours per week	32	12 [24–36]	24	6.6 [8–36]	24^b^	33 [7–40]

^a^For data of community nurses (*n* = 7) on the MAINtAIN-C Barriers, missing data of two respondents with one missing item was imputed. For data of team members (*n* = 69) on the MAINtAIN-C Barriers, missing data of three respondents were imputed (of whom one respondent had one missing item, one respondent had two missing items and one respondent had three missing items)

^b^Based on *n* = 67 due to missing data

^c^Based on *n* = 65 due to missing data

### Results per process component

We describe the results per component according to the framework of Saunders [17].

### Dose delivered, dose received exposure and reach

#### Community nurses

NitL was delivered as intended (dose delivered) since the systematic approach and training were undertaken. NitL was partly received as intended since all nurses developed implementation plans and engaged in group training, but only four actively used the background theory within the training (dose received exposure). The community nurses attended all planned plenary meetings (reach).

### Fidelity

#### Community nurses

In Additional file 4, Box 1, nurses’ planned implementation strategies are presented. According to the nurses, the strategies were implemented largely in practice, such as in coaching team members.

*Yes, I have had individual discussions. I have questioned team members, for example, you are responsible for this client, was your starting point client self-reliance and encouraging functional activities? I have asked people how this went, and were there things that were difficult. How can I help you with this? (Community nurse 6)*

Two strategies were not fully implemented as intended, namely providing information flyers to new clients and shadowing team members in practice. The review of implementation plans showed that all nurses developed a plan, however not all nurses described the evaluation of the plan and developed Specific, Measurable, Acceptable, Realistic and Time-bound (SMART) goals [11].

The review of patient records showed the degree to which diagnoses, interventions and outcomes related to encouraging ADL and IADL. Only a few nursing diagnoses, related to ADL, were described by community nurses and reported in patient records by team members. Nursing interventions and outcomes were described in over half of the included records by nurses, mainly for ADL, and reported in two out of three cases by team members. The results are reported in Table 3.

**Table 3:** Nursing diagnoses, interventions and outcomes regarding encouraging functional activities as described and reported in patient records (*n* = 28)

	Number of records (%) described	Number of records (%) reported
**Diagnoses**		
ADL	4 (14.3)	3 (10.7)
IADL	0 (0.0)	0 (0.0)
General activities	0 (0.0)	0 (0.0)
**Interventions**		
ADL	16 (57.1)	11 (39.3)
IADL	0 (0.0)	0 (0.0)
General activities	2 (7.1)	1 (3.6)
**Outcomes**		
ADL	15 (53.6)	10 (35.7)
IADL	0 (0.0)	0 (0.0)
General activities	0 (0.0)	0 (0.0)

#### Team members

Most team members acknowledged they were motivated by community nurses to encourage functional activities. Several members stated that they had been appointed as local opinion leaders by community nurses to lead the encouragement of functional activities in practice:

*In addition, the community nurse also appointed a group for the project, they conducted and steered the programme and they had regular meetings. Then, mail or messages were used to keep us informed. (Team member 2)*

Most of the members stated that encouraging functional activities became a fixed agenda item during team meetings. They also indicated that two nurses and a local opinion leader shadowed them during their daily work in practice:

*Yes, and the community nurse worked alongside everyone to see how we did it. This wasn’t for control, rather more to observe how we did it and afterwards to give tips. It was fine. (Team member 2)*

### Dose received satisfaction

#### Community nurses

All nurses were generally positive about the programme. They indicated that NitL made them more conscious about their routines, enabled team members to change practice and encouraged functional activities in older adults. They found the systematic approach and the training useful to further develop their role, and indicated that it was feasible to develop an implementation plan for themselves in future.

*Yes, what you must do was very clearly written, the steps were extensively described, so I found it a clear system in the way that it was written out. (Community nurse 7)*

However, opinions concerning the background theory varied. Three nurses indicated they did not consider the theory relevant for strengthening their leadership, whereas four nurses appreciated the information.

*Yes, I think that it was valuable, a little refresh [of] your memory about that part of the theory. (Community nurse 7)*

*Well, it didn’t make that much of an impression on me. (Community nurse 4)*

Some nurses suggested adapting the delivery of NitL by first providing the training, followed by the systematic approach, as well as providing more group training. Other recommendations were extending the implementation period, and implementing NitL within more community care teams in the organisation.

*Yes, but we could have given more input in the regions, it remained within our team. It was a missed opportunity to broaden the implementation. (Community nurse 4)*

They also recommended providing fewer examples of implementation strategies within the approach to leave more room for their own interpretation, simplifying the e-learning design, focusing more on motivational interviewing in the training, and shortening the MAINtAIN-C questionnaire. Nurses would also have liked the opportunity to collaborate with participating nurses from the other organisation.

#### Team members

The team members were positive about NitL, indicating that the content was in line with current practice and with the vision of their organisation. Most team members were satisfied with the coaching from community nurses and found clinical lessons educational.

*Then the community nurse said, occasionally, ‘Hey, you can try this, think about this or that’, and then I could consider it. Looking at things in that way is fine. (Team member 6)*

Some team members recommended extending clinical lessons after the implementation, and some members recommended better and more communication between the hospital setting and their organisations on encouraging functional activities of older adults.

### Context

#### Community nurses

Community nurses valued collaboration with other nurses and interventionists, as well as the facilitation of the organisation to be part of this research. A structured plan and the official status of NitL helped their team members encourage functional activities.

*I also see with my team that it really helps when they can follow an action plan, and not that once again something vague is dropped on them. (Community nurse 1)*

Nurses indicated that time constraints combined with a high complexity of care were barriers to implementing evidence. Another inhibiting factor was that not all team members were aware of the importance of encouraging activities in older adults. Some nurses also stated that the individual provision of community care (instead of in a team) was a barrier for enabling their team members to change practice.

*I find it difficult to check, you know, if the team members actually do that in practice because you don’t see that when you work in home care, you don’t see what someone says, tells, or asks a client. That makes it difficult. (Community nurse 3)*

The three strongest perceived barriers from the MAINtAIN-C Barriers scale were item 1, “clients are often able to perform ADLs more independently than they now do”(*M* = 6.29, *SD* = 2.43) and item 4, “family or informal caregivers expect the nurses and nurse assistants to take over the activities that clients themselves can still perform”(*M* = 5.71, *SD* = 2.06). Further, they experienced item 3, “clients ask for help with ADLs so that they can get extra attention” (*M* = 5.29, *SD* = 0.76) as a barrier.

#### Team members

According to team members, clear communication and structure, and agreements within the team were facilitators for encouraging functional activities of older adults. Barriers were a lack of time combined with high complexity of care and too few team meetings to discuss matters on encouraging activities as a group. They also acknowledged that expectations about receiving or providing care could be a hindrance in encouraging functional activities.

The two strongest barriers perceived by team members were the same as for community nurses, namely item 1 (*M* = 6.75, *SD* = 2.00) and item 4 (*M* = 5.64, *SD* = 1.56). They also experienced item 2 “clients are afraid to walk on their own, without help from others” (*M* = 5.39, *SD* = 1.40) as a barrier.

## Discussion

The results of this study show that the NitL programme was largely executed according to plan. NitL components were delivered and received, and all community nurses developed implementation plans, engaged in group training and attended plenary meetings. Most implementation strategies were realised; however, not all implementation plans were complete. Community nurses perceived NitL as useful and educational, and most team members were satisfied with coaching from the community nurses. For both nurses and team members, time constraints combined with a high complexity of care were barriers in practice, and that clients are often able to perform ADLs more independently than they now do. In contrast, clear communication and a structured plan were facilitators. For optimisation of the programme, community nurses recommended providing the training first, followed by the systematic approach, as well as providing more group training. The team members recommended continuing the provision of clinical lessons (an implementation strategy of the community nurses).

In our study, there was limited use by nurses of the background theory via the e-learning programme. This may be explained by the fact that not all community nurses in our study considered the background theory relevant. Previous research also indicates that e-learning is not effective on its own, but rather depends on the extent to which the content and its use are perceived as necessary [33]. Another explanation may be that instructions on using the background theory were not clear enough [33, 34]. For future implementations of NitL, it is necessary to give greater consideration to the goal, content and instructions of the background theory via the e-learning programme [33, 34].

Only a few community nurses in our study fully completed the implementation plans by developing SMART implementation goals and describing the evaluation of the plans. Although an interventionist experienced in implementation processes was available to support nurses during the development of the implementation plans, no prior training was given to increase skills and knowledge in developing such plans. As indicated during the interviews, it might be the case that a lack of time was a factor, or a lack of knowledge or skills, when designing and completing implementation plans. The studies by Mallion and Brooke [35] and Gifford et al. [36] support the view that the barriers perceived by community nurses during an implementation process are a lack of time, knowledge and skills. Hence, for future implementations of NitL, more training to increase skills and knowledge for developing an implementation plan is necessary. Moreover, as recommended by community nurses during interviews, group training should be provided first, followed by the systematic approach. Further, the description and reporting of nursing diagnoses, interventions and outcomes related to encouraging functional activities were mainly limited to ADL. An explanation could be that the community nurses and their team members primarily encourage ADL, as the provision of IADL care is more the responsibility of domestic workers [37].

### Strengths and limitations

Although nurse leadership improves high-quality community care, sound evaluation studies on programmes to empower nurse leadership remain scarce [38]. This process evaluation combined qualitative and quantitative methods and incorporated data from both community nurses and their team members from two long-term care organisations, which provided more profound insights into the implementation of the programme and increased trustworthiness of results. A limitation of the study may be that motivated community nurses have been overrepresented since convenience sampling was applied, which potentially may have led to a more positive evaluation of NitL. Further research is needed to determine whether our findings can be generalised to other community care teams. Another limitation was that the researcher who conducted the interviews was also involved as an interventionist, which may have led to socially desirable answers by community nurses and team members. However, respondents were informed about the anonymous treatment of data.

### Implications

The programme was perceived as worthwhile by community nurses and their team members. However, attention should be paid to adapting several aspects of the programme. First, more consideration should be given to the goal, content and blended perspective of the background theory via the e-learning programme. Second, we recommend adapting the delivery and content of the components of NitL. Group training should be extended and the training should be provided first, followed by the systematic approach. NitL can then be used to develop community nurse leadership in implementing evidence further to support the delivery of high-quality community care. Further research is needed to provide insights into the effects of NitL on community nurse leadership.

## Conclusion

In this study, the implementation of NitL into community care practice was evaluated. The programme consists of a systematic approach and training to 1) empower community nurses in implementing evidence, targeted at encouraging functional activities of older adults, and 2) train community nurses in enabling team members to change their practice. Our results indicated that NitL was largely executed as planned in practice. The systematic approach and training appear to strengthen the leadership of community nurses in systematically implementing evidence, and enabling team members to change practice. Adaptations to the programme are recommended, such as providing more training to community nurses. The programme can then be used to empower community nurse leadership in the community care setting, and support the provision of high-quality care.

## Supplementary Information

**Additional file 1.** Topic list with examples of the questions of the interviews.

**Additional file 2.** Example of the checklist for reviewing the implementation plans.

**Additional file 3.** Example of the checklist for the review of patient records.

**Additional file 4: Box 1.** The planned implementation strategies of the community nurses as described in their implementation plans.

## Data Availability

The datasets used and/or analysed during the current study are available from the corresponding author upon reasonable request.

## Abbreviations

ADL: Activities of daily living
GARS: Groningen Activity Restriction Scale
IADL: Instrumental activities of daily living
MAINtAIN-C: Maastricht Nurses Activities Inventory for Community Care
NitL: Nurses in the Lead
SMART: Specific, measurable, acceptable, realistic and time-bound
