# Supplementary material for: Process evaluation of a programme to empower community nurse leadership
Source: BMC Nurs. 2021 Jul 12;20:127. doi: 10.1186/s12912-021-00650-y (PMC8273989; doi:10.1186/s12912-021-00650-y)
Supplement: Supplementary file 1 — Additional file 1. Topic list with examples of the questions of the interviews. [file 12912_2021_650_MOESM1_ESM.pdf]

Additional files

Additional file 1. Topic list with examples of the questions of the interviews

Topics	Examples of questions from the interviews with community nurses
Fidelity	• Have you executed the strategies from the implementation plan to implement evidence for encouraging functional activities?<br>If yes, can you amplify your answer?
Dose delivered	• Were you able to open the systematic approach via the e-learning programme?<br>If yes, can you name some of the depicted content?
Dose received exposure	• Did you use the background theory of the systematic approach via the e-learning programme?<br>If yes, can you amplify your answer?
Dose received satisfaction	• How satisfied were you with the NitL programme?<br>Can you amplify your answer?
Context	• Did you experience any facilitators in developing the implementation plan?<br>If yes, what were these facilitators?
**Topics**	**Examples of questions from the focus groups with team members**
Fidelity	• Did the community nurse of your team enable you to change your practice in encouraging functional activities of older adults?<br>If yes, can you amplify your answer?
Dose received satisfaction	• How satisfied were you with the coaching you received from the community nurse of your team?
Context	• Did you experience any barriers in encouraging functional activities of older adults?<br>If yes, what were these barriers?
**Topics**	**Examples of questions from the interviews with older adults and their informal caregivers**
Fidelity	• Did the nurses encourage you to perform functional activities independent?<br>If yes, can you amplify your answer?
Dose received satisfaction	• How satisfied were you with how the nurses encouraged you (or did not encourage you) to independently perform functional activities?
Context	• Did you experience any barriers in independently performing functional activities?<br>If yes, what were these barriers?
