# Supplementary material for: Process evaluation of a programme to empower community nurse leadership
Source: BMC Nurs. 2021 Jul 12;20:127. doi: 10.1186/s12912-021-00650-y (PMC8273989; doi:10.1186/s12912-021-00650-y)
Supplement: Supplementary file 2 — Additional file 2. Example of the checklist for reviewing the implementation plans. [file 12912_2021_650_MOESM2_ESM.pdf]

Additional file 2. Example of the checklist for reviewing the implementation plans

Name	Requirements	Yes	No	Comments
**Step 1: Formulate a proposal for change and SMART (Specific, Measurable, Attainable, Relevant, Time-Bound) goals.**	A. Has it described what must change? *(A)*<br>B. Has it described how much must change? *(B)*<br>C. Has it described who must change? *(C)*<br>D. Has it described when the change must occur? *(D)*<br>E. Has a SMART goal been formulated based on A, B, C and D?	X<br>X<br>X<br>X<br>X		
**Step 2: Assess the performance of professionals in encouraging functional activities and related barrier and formulating SMART targets for change.**	A. Has the MAINtAIN-C questionnaire been administered?<br>B. Has the Excel tool been completed?<br>C. Has it described the current performance of professionals in encouraging functional activities?<br>D. Has it described the barriers of professionals in encouraging functional activities?<br>E. Have SMART goals been formulated based on current performance and barriers?	X<br>X<br>X<br>X	X (E)	The goals are not formulated SMART.
**Step 3: Select and tailor strategies.**	A. Have the implementation strategies been described?<br>B. Has it described how the strategies are tailored to the barriers?<br>C. Has it described who is responsible for executing the strategies?	X<br>X<br>X		
**Step 4: Plan the implementation process.**	A. Has it described who is going to execute the implementation of the strategies in practice?<br>B. Has it described who is involved during the implementation?<br>C. Has it described how much and when the strategies are executed?	X<br>X<br>X		
**Step 5: Integrate the improvement within practice.**	A. Has it described which actions need to ensure that the change is secured?<br>B. Has it specifically described who is executing these actions and when?	X<br>X		
**Step 6: Evaluate and revise the plan.**	A. Has it described which actions are undertaken to evaluate the implementation process?<br>B. Has it specifically described who is executing these actions and when?	X<br>X		
