# Supplementary material for: Process evaluation of a programme to empower community nurse leadership
Source: BMC Nurs. 2021 Jul 12;20:127. doi: 10.1186/s12912-021-00650-y (PMC8273989; doi:10.1186/s12912-021-00650-y)
Supplement: Supplementary file 3 — Additional file 3. Example of the checklist for the review of patient records. [file 12912_2021_650_MOESM3_ESM.pdf]

Additional file 3. Example of the checklist for the review of patient records

Patient number: X	*Are the diagnoses, outcomes or interventions related to encouraging functional activities (ADL, IADL, general activities*) reported within the patient record?*			*Are the diagnoses, outcomes or interventions, related to encouraging functional activities (ADL, IADL, general activities*) reported within the patient record, also reported?*		
**Nursing process**	**Yes**	**Description**	**No**	**Yes**	**Description**	**No**
**ADL**						
Diagnose			X			X
Outcome	X	Within two months, Mr X is able to independently put on and take off his compression stockings with the use of an assistive device.		X	‘Mr X did put on his compression stockings this morning, it went well’. ‘Mr X still needs small steering but has put on the stockings completely independent’. ‘Mr X is satisfied with the assistive device and its use’.	
Intervention			X			X
**IADL**						
Diagnose			X			X
Outcome			X			X
Intervention			X			X
**General activities**						
Diagnose			X			X
Outcome			X			X
Intervention			X			X

**We followed the clustering of the ADL (e.g. using the toilet) and IADL (e.g. preparing breakfast) as presented in the Groningen Activity Restriction Scale (GARS) developed to measure disability (28). For the definition of general activities (e.g. discussing with clients which activities they can still perform), we followed the activities as presented in the MAINtAIN-C Behaviours scale (15).*
