# Supplementary material for: Process evaluation of a programme to empower community nurse leadership
Source: BMC Nurs. 2021 Jul 12;20:127. doi: 10.1186/s12912-021-00650-y (PMC8273989; doi:10.1186/s12912-021-00650-y)
Supplement: Supplementary file 4 — Additional file 4: Box 1. The planned implementation strategies of the community nurses as described in their implementation plans. [file 12912_2021_650_MOESM4_ESM.pdf]

Additional file 4. Box 1. The planned implementation strategies of the community nurses as described in their implementation plans

Box 1. The planned implementation strategies of the community nurses as described in their implementation plans

Implementation strategies

- Different content and structure of team meetings: using case studies (discussing the process of encouraging functional activities for particular older adults), emphasising the use of informative media, sharing experiences and giving each other feedback
- Individual coaching (to share experiences with team members and encourage the provision of giving feedback)
- Providing information flyers to new clients on how to independently perform functional activities
- Having a conversation with the manager and the board of the organisation to discuss the development of information flyers, which can be provided to new clients on how to independently perform functional activities
- Providing a newsletter within the organisation about the programme and the importance of encouraging functional activities
- Providing clinical lessons to team members (together with an occupational therapist and physiotherapist about the importance of encouraging independent performance of functional activities and the use of assistive devices and with a mental health care worker to discuss mental problems of clients in relation to resistance to change)
- Having a conversation with the manager and the board of the organisation to provide clarity on the job description of community nurses related to their leadership role
- Appointing team members as opinion leaders (to lead by example, to motivate, inform and prioritise the encouragement of functional activities)
- Shadowing their team members during the provision of community care in practice (a community nurse, occupational therapist or opinion leaders should go along with the other team members to increase awareness, to learn from each other and to give feedback)
